# High-Yield Expressed Human Ferritin Heavy-Chain Nanoparticles in *K. marxianus* for Functional Food Development

**DOI:** 10.3390/foods13182919

**Published:** 2024-09-15

**Authors:** Xinyi Lu, Liping Liu, Haibo Zhang, Haifang Lu, Tian Tian, Bing Du, Pan Li, Yao Yu, Jungang Zhou, Hong Lu

**Affiliations:** 1 State Key Laboratory of Genetic Engineering, School of Life Sciences, Fudan University, Shanghai 200438, China; 21110700035@m.fudan.edu.cn (X.L.); 22260700019@m.fudan.edu.cn (L.L.); 22210700122@m.fudan.edu.cn (H.L.); 20210700047@fudan.edu.cn (T.T.); yaoyu@fudan.edu.cn (Y.Y.); 2 Shanghai Engineering Research Center of Industrial Microorganisms, Fudan University, Shanghai 200438, China; 3 North America Nutrition Research and Development Society, Guangzhou Aoungo Biotech Co., Ltd., Guangzhou 510310, China; haibo.zhang@aoungo.com; 4 College of Food Science, South China Agricultural University, Guangzhou 510642, China; dubing@scau.edu.cn (B.D.); lp19900815@scau.edu.cn (P.L.)

**Keywords:** recombinant ferritin, nanoparticle, *Kluyveromyces marxianus*, functional foods

## Abstract

The use of Generally Recognized as Safe (GRAS)-grade microbial cell factories to produce recombinant protein-based nutritional products is a promising trend in developing food and health supplements. In this study, GRAS-grade *Kluyveromyces marxianus* was employed to express recombinant human heavy-chain ferritin (rhFTH), achieving a yield of 11 g/L in a 5 L fermenter, marking the highest yield reported for ferritin nanoparticle proteins to our knowledge. The rhFTH formed 12 nm spherical nanocages capable of ferroxidase activity, which involves converting Fe^2+^ to Fe^3+^ for storage. The rhFTH-containing yeast cell lysates promoted cytokine secretion (tumor necrosis factor-α (TNF-α), interleukin-6 (IL-6), and -1β (IL-1β)) and enhanced locomotion, pharyngeal pumping frequency, egg-laying capacity, and lifespan under heat and oxidative stress in the RAW264.7 mouse cell line and the *C. elegans* model, respectively, whereas yeast cell lysate alone had no such effects. These findings suggest that rhFTH boosts immunity, holding promise for developing ferritin-based food and nutritional products and suggesting its adjuvant potential for clinical applications of ferritin-based nanomedicine. The high-yield production of ferritin nanoparticles in *K. marxianus* offers a valuable source of ferritin for the development of ferritin-based products.

## 1. Introduction

Ferritin is a ubiquitous protein found in most organisms, including animals, bacteria, and plants, but it is absent in yeasts [1]. In mammals, ferritin is composed of two distinct subunit types: the H subunit (21 kDa) and the L subunit (19 kDa). The H subunit contains a ferroxidase center oxidizing Fe^2+^ to Fe^3+^, which is crucial for rapid detoxification and intracellular iron transport [2]. Ferritin nanoparticles, co-assembled by 24 subunits, form a highly symmetrical structure with an outer diameter of around 12 nm and an inner diameter of about 8 nm, and can accommodate up to approximately 4500 iron atoms in their cavity [3]. Ferritin plays a pivotal role in maintaining iron homeostasis by regulating the storage and release of iron, storing it in an insoluble, non-toxic form while converting it into a soluble form to ensure its intracellular bioavailability [4]. Ferritin nanoparticles display outstanding stability even at high temperatures (>80 °C) and in the presence of various denaturants [5]. Moreover, ferritin nanoparticles can reversibly disassemble in extremely acidic (pH 2–3) or alkaline (pH 10–12) conditions and reassemble when the pH returns to 7.0 [6].

The unique properties of its self-assembly, stable nanocage structure, and ferroxidase activity make ferritin a favored carrier in nanotechnology [7]. Particularly in the medical field, ferritin has been widely studied for its applications. Utilizing the encapsulation capacity of its nanocage cavity and tumor-targeting properties of ferritin, it has been used to deliver chemotherapy drugs such as doxorubicin, cisplatin, and carboplatin, in addition to siRNA for tumor immunotherapy [8,9,10]. Ferritin also carries imaging agents (e.g., fluorescence dyes, radioisotopes, and MRI contrast agents) to visualize tumor tissues and track tumor progression [11]. Additionally, ferritin is under exploration for novel vaccine development, as the 24-subunit nanocage could present a repetitive array of antigens for improving immunogenicity and enhancing protective responses [12]. Despite the wide application of ferritin nanoparticle technology in medical research fields [13], studies focusing on the intrinsic biological functions of ferritin itself, especially for clinical use, remain scarce.

Early studies primarily obtained ferritin through the extraction and purification from horse spleen, while microbial cell factory technologies have been extensively used for the recombinant production of various ferritins, including human ferritin heavy-chain (hFTH), human ferritin light-chain (hFTL), and *Helicobacter pylori* ferritin (Hpfer). Among these, hFTH has been successfully expressed in various hosts, including *Escherichia coli* (*E. coli*), *Pichia pastoris*, and *Hansenula polymorpha*, with yields of 7.26 g/L [14], 25.7 mg/L [15], and 1.9 g/L [16], respectively. The yield of hFTL expressed in *E. coli* was 10 mg/L [17], typically as inclusion bodies. The expression of Hpfer in a baculovirus-insect cell expression system resulted in a yield of 11.25 mg/L [18]. These recombinant ferritins have demonstrated excellent biocompatibility in drug delivery for cancer therapy [19]. Exploring more efficient and higher-yielding technologies, with safer host cells for ferritin production, will facilitate the development of ferritin-based products and their application in medical and health care.

*Kluyveromyces marxianus* (*K. marxianus*) is a GRAS-grade yeast that has shown great potential for industrial applications in the expression of recombinant proteins, including enzymes [20,21,22], and self-assembled virus-like particles [23,24,25]. The yield of these virus-like nanoparticles in *K. marxianus* exceeds that of other expression systems. In this study, we aimed to achieve the high-yield production of human ferritin heavy-chain (hFTH) nanoparticles in GRAS-grade *Kluyveromyces marxianus* and evaluate their potential for functional food development. We successfully recombinantly expressed hFTH (rhFTH), obtaining a remarkable soluble yield of 11 g/L in a 5 L fermenter, which, to our knowledge, is the highest yield reported for human ferritin nanoparticles. Transmission electron microscopy (TEM) analysis revealed that the rhFTH self-assembled into spherical cage-like nanoparticles with a diameter of approximately 12 nm. It was able to catalyze the conversion of Fe^2+^ to Fe^3+^, consistent with native ferritin. Excitingly, the rhFTH-containing yeast cell lysates promoted cytokine secretion (IL-6, IL-1β, TNF-α) and enhanced locomotion, pharyngeal pumping frequency, egg-laying capacity, and lifespan under heat and oxidative stress in the RAW264.7 mouse cell line and the *C. elegans* model, respectively, while yeast cell lysate alone had no such effects. The rhFTH itself demonstrated immune-boosting and vitality-enhancing functions, suggesting that rhFTH nanoparticles not only serve as a ferritin-based nanocage carrier for drug and vaccine development but also offer an immune adjuvant for clinical applications of ferritin-based medicines. Additionally, rhFTH from *K. marxianus* also provides scientific support and shows potential for the development of ferritin-based novel food and nutritional products.

## 2. Materials and Methods

### 2.1. Construction of a Kluyveromyces marxianus Strain Expressing Recombinant Human Ferritin Heavy-Chain (KM-rhFTH)

The h*FTH1* gene encoding human ferritin heavy-chain (UniProtKB: P02794) was optimized for *K. marxianus* codon usage and synthesized in full length (GENEWIZ, Suzhou, China). The *FTH1* gene was amplified using primer pairs 125-FTH1-F (5′-TTTTTTGTTAGATACTAGTCCCGGGATGACTACCGCTTCCACTTCTCAA-3′) and 125-FTH1-R (5′-AGCTTGCGGCCTTAAGCGGCCGCTTAGCTTTCGTTGTCACTATCACCC-3′). The amplified fragment was ligated into the Sma I /Not I linearized pUKDN125 vector by Gibson assembly and then transformed into *K. marxianus* strain T1 *Ura3*Δ *Atg1*Δ [26]. Transformants were screened on synthetic defined (SD) plates (0.67% yeast nitrogen base, 2% glucose, and 2% agar). A positive clone verified by PCR and sequencing was designated to the KM-rhFTH strain. As a control, the empty pUKDN125 vector was also transformed into *K. marxianus* strain T1 *Ura3*Δ *Atg1*Δ to generate the KM strain.

### 2.2. Fermentation of KM-rhFTH Strain

Fermentation in shake flasks: The freshly grown KM-rhFTH strain on YPD solid medium was inoculated into 150 mL shake flasks containing 50 mL YG medium (2% yeast extract, 4% glucose), with an initial OD_600_ of 0.1, and cultured at 30 °C and 220 rpm for 72 h, followed by examinations of the rhFTH expression in *K. marxianus*.

Fermentation in fermenters: 1.5 L mineral medium was prepared in a 5 L fermenter (BXBIO fermenter, Shanghai, China) [27]. Next, 150 mL seed culture of KM-rhFTH, grown in SM medium ((NH_4_)_2_SO_4_ 5 g/L, Glucose 10 g/L, MgSO_4_·7H_2_O 0.5 g/L, KH_2_PO_4_ 3 g/L, trace elements 2 mL/L, vitamins 1 mL/L, citric acid/K_2_HPO_4_ buffer 200 mM), was cultivated for 16 h until it reached an OD_600_ of 10 and then inoculated into the 1.5 L mineral medium. The fermenter was maintained at 30 °C, with the pH automatically controlled at 5.5 using ammonium hydroxide, and the dissolved oxygen levels were maintained at approximately 30%, 10%, and 0%, respectively, corresponding to different glucose feeding rates from 6 h to 72 h, as described previously [23]. The final total glucose addition varied based on the fermentation conditions. The fermentation time was 72 h, and the cell cultures were used for the subsequent purification experiment.

### 2.3. Analysis of rhFTH

Cell lysis: 1 mL of the yeast cell cultures from shake-flask fermentation (approximately OD_600_ = 30) was collected by centrifugation at 8000 rpm for 5 min. The yeast cells were washed once with phosphate-buffered saline (PBS, 137 mM NaCl, 2.7 mM KCl, 10 mM Na_2_HPO_4_, 1.8 mM KH_2_PO_4_, pH 7.4), and then resuspended in 500 μL of PBS. Cells were disrupted with 400 μL of glass beads using a Bead-beater (FastPrep-24, MP, Santa Ana, CA, USA) at 6 m/s for 2 min, followed by centrifugation at 14,000 rpm for 20 min. Yeast cells of the KM-rhFTH strain from fermenter fermentation were suspended in PBS at an OD_600_ = 100 and disrupted by high-pressure homogenization on a JN-02C Homogenizer (JNBIO, Guangzhou, China) to obtain the cell lysate of KM-rhFTH strain.

SDS-PAGE and Western blot analysis: The supernatant and precipitation of cell lysate were analyzed using 15% SDS-PAGE. A Western blot analysis was performed using ferritin heavy-chain rabbit monoclonal antibodies (1:1000 dilution; ABclone, Wuhan, China) and a goat anti-rabbit IgG secondary antibody HRP (1:5000 dilution; Abmart, Shanghai, China).

Protein quantification: Lactoglobulin, with a molecular weight of 18.4 kDa, similar to that of rhFTH, was prepared at concentrations of 100, 200, 300, 400, and 500 μg/mL to serve as a standard protein. The content of rhFTH was determined by scanning the bands of standard protein and rhFTH through grayscale scanning using GenoSens software v2150 (Clinx Science, Shanghai, China).

### 2.4. Orthogonal Design Experiment

An L_9_ (3^3^) orthogonal array was designed to optimize the rhFTH expression in shake flasks using SM medium. Nine parallel conditional experiments were set up with different concentrations of (NH_4_)_2_SO_4_ (5, 12, 18 g/L), glucose (10, 40, 60 g/L), and MgSO_4_·7H_2_O (0.5, 7, 10.5 g/L). Each experiment was run in triplicate. The relative soluble expression of the rhFTH was determined by SDS-PAGE and grayscale scanning as described above. The orthogonal array was analyzed using range analysis to evaluate the impact of each factor on ferritin yield.

### 2.5. Transmission Electron Microscope (TEM) Analysis

Ferritin nanoparticles were observed by the Tecnai G2 F20 S-Twin microscope. The sample was adsorbed on a carbon-coated copper grid for 30 s at room temperature and then dried using filter paper. After negative staining with 2% (*w*/*v*) aqueous uranyl acetate for 2 min, the grid was imaged under an acceleration voltage of 200 kV.

### 2.6. Purification of rhFTH

The cell lysate of KM-rhFTH strain was heated at 75 °C for 10 min, followed by centrifugation at 15,000 rpm for 30 min at 4 °C. The heat-treated supernatant was then subjected to ion exchange chromatography (IEC) using a DEAE Bestarose FF column (Bestchrom, Zhejiang, China), and eluted with 20 mM PB (20 mM Na_2_HPO_4_, 20 mM NaH_2_PO_4_, pH 7.0), 0.6 M NaCl. Fractions were analyzed by 15% SDS-PAGE. The purified rhFTH was also examined by 8% native-PAGE using the human ferritin heavy-chain produced by *E. coli* (HY-P70246, MedChemExpress, Shanghai, China) as a reference, and was quantified by the BCA Protein Assay Kit (Sangon Biotech, Shanghai, China) using bovine serum albumin as standard.

### 2.7. Measurement of Iron Uptake and Release Kinetics

Iron-uptake measurement: Reactions were conducted in 100 µL of 100 mM sodium acetate buffer (pH 5.5) supplemented with or without 0.3 mg/mL rhFTH. Iron uptake reactions were initiated by addition of 50 µL of 0.25, 0.5, or 1.0 mM FeSO_4_ solution. The absorbances at 310 nm were measured by an Eon™ High Performance Microplate Spectrophotometer at an interval of 2 s (BioTek, Winooski, VT, USA) [28]. The ΔA_310_ values were the absorbances of the rhFTH against the control (without rhFTH) with corresponding concentrations of FeSO_4_ added.

Iron-binding capacity: 0.3 mg/mL rhFTH in 100 mM sodium acetate buffer (pH 5.5) was incubated with an equal volume of 0.1, 0.25, 0.5, 1.0, or 5.0 mM FeSO_4_ solution for 2 h. After centrifugation for 10 min, the supernatants were collected and ultrafiltered with 30 kD Amicon^®^ Ultra (Merck, Burlington, MA, USA) five times to remove the free iron. The iron content was determined by inductively coupled plasma mass spectrometry (ICP-MS) at Xinhui Nengji (Shanghai, China) Technology Co., Ltd.

Iron-release measurement: Kinetic measurement of iron release was performed according to previous methods [29]. Briefly, 50 μL of 2 mM ferrozine and 50 μL of 2% thioglycolic acid in 0.5 M sodium acetate buffer were incubated with 100 μL of the rhFTH treated by 1 mM FeSO_4_ above; meanwhile, the same concentration of the rhFTH without FeSO_4_ treated was used as the blank control. The absorbance at 562 nm was measured at an interval of 5 s. ΔA_562_ represented the difference between the FeSO_4_-treated rhFTH and the control.

### 2.8. Cell Proliferation and Cytokine Assays

For the cell proliferation assay, about 2 × 10^3^ mouse macrophage RAW264.7 cells at the exponential phase were inoculated in 100 μL/well of DMEM medium (89% DMEM basal medium, 10% fetal bovine serum, 1% antibiotics) and incubated at 37 °C for 24 h. After centrifuging and discarding the supernatant, 100 μL of *K. marxianus* cell lysate with or without rhFTH, and media were added and incubated at 37 °C for 24 h, respectively. *K. marxianus* cell lysates with or without rhFTH were diluted with culture media and then sterilized by filtration. Subsequently, cells were washed three times with preheated PBS, and then 100 µL of 10% CCK-8 culture medium was added to each well and incubated for additional 4 h. The absorption at 450 nm was determined using the Eon™ Spectrophotometer (BioTek, Winooski, VT, USA).

For the cytokine assays, RAW264.7 cells were cultured in 6-well plates containing 1 mL of medium per well. Approximately 2 × 10^4^ cells at the exponential phase were inoculated and incubated at 37 °C for 24 h. Then, cells were harvested by centrifugation. Next, 1 mL amounts of the same concentrations of *K. marxianus* cell lysate with or without rhFTH, 1 ug/L LPS, and media were added and incubated at 37 °C for 24 h, respectively. Supernatants of the cell cultures were collected to determine the contents of IL-6, IL-1β, and TNF-α using the ELISA kits (Meimian Industrial, Yancheng, China). Six replicates were set up for each experimental group.

### 2.9. Experimental Nematodes, Culture, and Lifespan Analysis

Wild-type N2 *C. elegans* strain (*Caenorhabditis elegans* Genetics Center, USA) was cultured on nematode growth medium (NGM) (NaCl 3 g/L, tryptone 2.5 g/L, streptomycin sulfate 0.2 g/L, 1 M CaCl_2_ 1 mL, 1 M MgSO_4_ 1 mL, 1 M potassium phosphate buffer 25 mL, 5 mg/mL cholesterol 1 mL) in 55 mm Petri dishes at 20 °C, with *E. coli* OP50 bacteria as a food source, and synchronized as described previously [30].

The synchronized *C. elegans* were randomly divided into a blank group, two ferritin groups, and two corresponding control groups with 30 nematodes per plate for each group. *K. marxianus* cell lysates with or without rhFTH were diluted with *E. coli* OP50 broth and then sterilized by filtration. All samples were then spread onto the surface of the NGM plate and cultured in an artificial climate chamber at 20 °C and transferred to fresh NGM plates every day for the first 7 days, followed by every 2 days thereafter. Meanwhile, the counts of dead and alive nematodes were recorded until all nematodes died. Survival curves were plotted based on the observed survival and mortality data of the nematodes.

A heat stress assay was performed by incubating the nematodes at 37 °C. For the oxidative stress assay, the nematodes were transferred to fresh NGM plates supplemented with 10 μL of 30% hydrogen peroxide per 10 mL of NGM. The count of alive nematodes was recorded every hour until all nematodes died, and survival curves were plotted based on the observed survival and mortality data of the nematodes.

### 2.10. Locomotion, Pharyngeal Pumping, and Egg-Laying Assay

The locomotion ability of nematodes was assessed by recording the ratio of nematodes that moved spontaneously without touch stimulation within 30 s, which was conducted simultaneously with the lifespan analysis. For the pharyngeal pumping assay, synchronous L4-stage nematodes were transferred to fresh culture plates with 20 nematodes per plate for each group. After cultivation for 5 days, 10–15 nematodes were randomly chosen from each group, and their pharyngeal pumping frequency was observed for 1 min using a microscope by gently touching their bodies with a platinum wire.

For the egg-laying assay, 10 nematodes in L4 stage were selected onto the culture medium of each group, with 1 nematode placed on each plate. The nematodes were transferred to new plates every 24 h, and the nematodes stopped laying eggs after 4–5 times. All the plates were maintained in the incubator for further culture. After the offspring nematodes developed, the number of nematodes before entering the spawning period (incubation at 20 °C for 24 h) was counted. The total number of eggs produced by each nematode in 6 plates was the spawning number of the nematode.

### 2.11. Analysis of T-SOD, CAT, ROS, and Lipofuscin in C. elegans

The nematodes in different groups were cultured for 5 days using the same methods as the lifespan assay. They were collected and washed three times with M9 buffer. After freezing and grinding, the mixture was centrifuged to discard the supernatant, resulting in a final preparation of 5% nematode homogenate. The activities of T-SOD and CAT were measured according to the relevant assay kits, with standardization based on the protein concentration of the supernatant.

The nematodes in different groups were cultured for 3 days and collected and washed three times with M9 buffer. After freezing and grinding, 50 μL of supernatant and 50 μL of H2DCF-DA were mixed in the dark, the fluorescence intensity was measured every 10 min for 2 h using a multifunctional microplate reader with an excitation wavelength of 485 nm and an emission wavelength of 530 nm, and the relative fluorescence intensity was standardized based on the protein concentration. Similarly, after 3 days, nematodes were anesthetized with 5% ether and then transferred to the 2% agarose gel pad. The fluorescence images were obtained through a fluorescence microscope (excitation wavelength 365 nm, emission wavelength 420 nm), a monochrome digital camera, and the Image Pro Plus software package (Version 6.0).

### 2.12. Statistical Analysis

Statistical analysis was performed using GraphPad Prism 8 software. All p-values were calculated using an unpaired two-tailed Student’s *t*-test, which was considered significant if the value was less than 0.05.

## 3. Results

### 3.1. Expression of Recombinant Human Ferritin Heavy-Chain in K. marxianus

**Figure 1:**
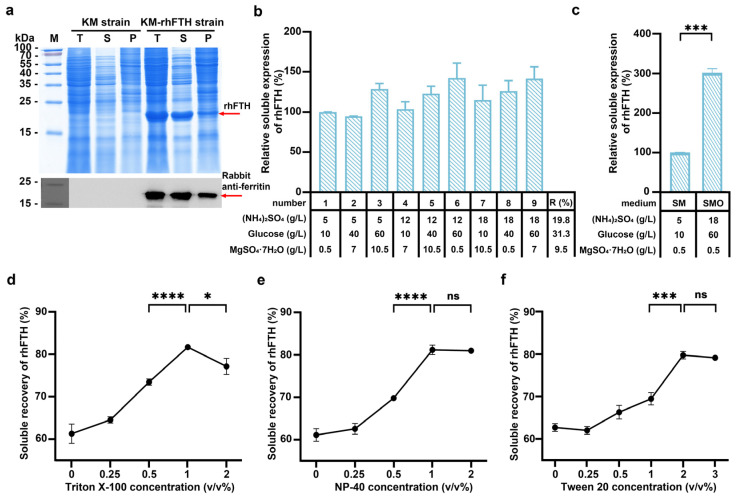
Expression of rhFTH in *K. marxianus*. (**a**) SDS-PAGE and Western blot analyses of rhFTH expression in *K. marxianus*. The KM and KM-rhFTH strains are *K. marxianus* transformed with pUKDN125 and pUKDN125-rhFTH, respectively. The red arrow highlights the bands of rhFTH. Rabbit anti-ferritin monoclonal antibody and secondary antibody goat anti-rabbit IgG were used for Western blot. M: PageRuler prestained protein ladder; T: total cell lysate; S: supernatant of cell lysate; P: precipitate of cell lysate. (**b**) An orthogonal design with three factors at three levels, including (NH_4_)_2_SO_4_ (5, 12, 18 g/L), glucose (10, 40, 60 g/L), and MgSO_4_·7H_2_O (0.5, 7, 10.5 g/L), was used to test the effects on soluble expression of rhFTH in SM medium in shake flasks at 220 rpm at 30 °C for 72 h. The soluble expression of rhFTH under different conditions was separately compared with that in SM medium. R: Range values of orthogonal design experiments. (**c**) Comparison of the soluble expressions of rhFTH in SM medium and the optimized SMO medium. (**d**–**f**) Effects of different concentrations of Triton X-100, NP-40, and Tween 20 in cell lysis solution on the recovery of soluble rhFTH. Statistical differences were analyzed using *t*-tests. * *p* < 0.05; *** *p* < 0.001; **** *p* < 0.0001; *p* > 0.05 (not significant, ns).

The recombinant KM-rhFTH strain was cultured in YG medium in shake flasks at 220 rpm for 72 h. Cells were collected and lysed, and the total cell lysate, supernatant, and precipitate were analyzed separately by SDS-PAGE and Western blot for rhFTH expression. As shown in Figure 1a, compared to the control strain, an additional 21 kDa protein band appeared in the total cell lysate, supernatant, and precipitate, consistent with the theoretical molecular weight of hFTH, and was identified using a rabbit anti-ferritin monoclonal antibody. A grayscale scanning analysis indicated that the soluble rhFTH comprised about 67% of the total rhFTH produced in *K. marxianus* (Figure 1a).

To improve the soluble expression of rhFTH in *K. marxianus*, an orthogonal design experiment involving three factors at three levels was conducted to optimize the components of the SM medium. The KM-rhFTH strain was then cultured in the designed medium containing different concentrations of glucose, (NH_4_)_2_SO_4_, and MgSO_4_·7H_2_O, and the soluble expression of rhFTH was analyzed by SDS-PAGE and grayscale scanning (Figure 1b). As a result, glucose was identified as the most significant factor, followed by (NH_4_)_2_SO_4_ and MgSO_4_·7H_2_O. With the optimized medium, named SMO, that consisted of 60 g/L glucose, 18 g/L (NH_4_)_2_SO_4_, and 0.5 g/L MgSO_4_·7H_2_O, the soluble expression of rhFTH was increased by 200% when compared to the SM medium (Figure 1c).

Furthermore, the effects of three detergents (Triton X-100, Tween 20, and NP-40) on the recovery of soluble rhFTH during cell lysis were tested. As shown in Figure 1d–f, the recovery rate increased with the tested concentrations up to 1% for Triton X-100, 1% for NP-40, and 2% for Tween 20, with a maximum increase of approximately 20%. Further increases in detergent concentrations did not enhance recovery rates (Figure 1e,f), and even decreased in the case of Triton X-100 (Figure 1d). Ultimately, it appears that the addition of 1% Triton X-100, 1% NP-40, or 2% Tween 20 in the cell lysis solution resulted in a relative increase in recovery rate by approximately 20% (Figure 1d–f).

### 3.2. Fed-Batch Fermentation of KM-rhFTH in 5 L Fermenters

**Figure 2:**
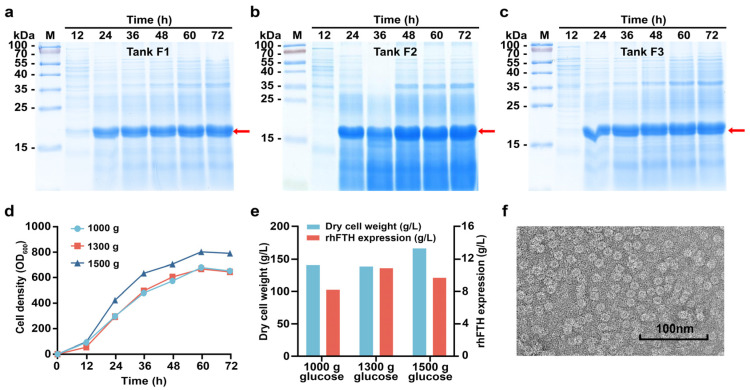
Fermentation of KM-rhFTH strain and production of rhFTH in 5 L fermenters. (**a**–**c**) The fermentation was carried out in three 5 L fermenters fed with 1000 g (Tank F1), 1300 g (Tank F2), and 1500 g (Tank F3) of glucose. Cells were collected every 12 h, lysed after a 5-fold dilution, then subjected to SDS-PAGE analyses for the expression of soluble rhFTH. (**d**) The growth curves of KM-rhFTH strain in the three fermenters fed with different amounts of glucose. (**e**) Cell dry weights at 60 h and soluble yields of rhFTH at 72 h in the three fermenters. (**f**) Observation of rhFTH in the supernatant of the cell lysate collected from Tank F2 at 72 h by transmission electron microscopy. Bar, 100 nm.

Given the significant impact of glucose on rhFTH expression observed in the orthogonal experiments conducted in flasks, parallel experiments were performed using three 5 L fermenters with total glucose supplementation of 1000 g (Tank F1), 1300 g (Tank F2), and 1500 g (Tank F3), respectively (Figure 2a–c). Glucose was fed from 6 h to 72 h of fermentation. The dissolved oxygen levels during fermentation were controlled at approximately 30%, 10%, and 0%, respectively. Among the three fermenters, cell growth rate was highest in F3, reaching an OD_600_ of 805 and a cell dry weight of 168 g/L at 60 h. The cell growth rates in Tanks F1 and F2 were quite similar, with cell dry weights reaching 140 g/L at 60 h (Figure 2d,e). At 72 h, the soluble rhFTH reached 11.0 g/L in F2, 9.8 g/L in F3, and 8.3 g/L in F1, respectively. A TEM analysis further revealed that the soluble rhFTH expressed in *K. marxianus* could self-assemble into ferritin nanoparticles with a diameter of approximately 12 nm (Figure 2f), which is consistent with the reported structure of native human ferritin.

### 3.3. Characterization of rhFTH Expressed in K. marxianus

**Figure 3:**
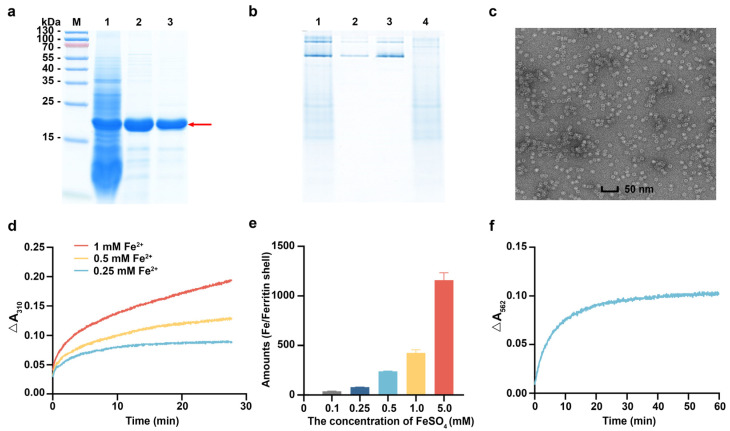
Purification and characterization of rhFTH produced by *K. marxianus*. (**a**) SDS-PAGE analysis of rhFTH purified by heat treatment coupled with DEAE chromatography. Lane M: PageRuler prestained protein ladder; Lane 1: Cell lysate supernatant of the KM-rhFTH; Lane 2: Heat-treated supernatant at 75 °C for 10 min; Lane 3: Elution fraction from DEAE column. (**b**) Native-PAGE. Lane 1: Cell lysate supernatant of the KM-rhFTH strain; Lane 2: Commercial recombinant ferritin expressed in *E. coli*; Lane 3: Purified rhFTH expressed in *K. marxianus*; Lane 4: Cell lysate supernatant of the *K. marxianus* host strain. (**c**) TEM analysis of purified rhFTH. Scale bar: 50 nm. (**d**) Iron uptake of the purified rhFTH. The experiments were carried out in solutions containing a fixed concentration of 0.3 mg/mL rhFTH and 0.25, 0.5, and 1 mM FeSO_4_, respectively. The reaction was performed at room temperature for a total of 30 min, and absorbance values at A_310_ nm were obtained every 2 s. The initial absorbance was subtracted to obtain ΔA_310_ nm. (**e**) Analysis of iron content in rhFTH by ICP-MS. (**f**) Analysis of iron release from rhFTH by the ferrozine method. Incubation with FeSO_4_ was conducted at a concentration of 1 mM.

To evaluate the iron-loading kinetics of rhFTH expressed in *K. marxianus*, the rhFTH was purified. The cell lysate supernatant of KM-rhFTH strain was heated at 75 °C for 10 min to remove some impurities. The grayscale scanning analysis showed that the purity of rhFTH in the supernatant reached 75% (Figure 3a, lane 2). Subsequently, rhFTH with 90% purity was obtained through DEAE chromatography (Figure 3a, lane 3). The purified rhFTH expressed in *K. marxianus* exhibited the same electrophoretic profile as commercial recombinant ferritin expressed in *E. coli* (Figure 3b, lanes 2 and 3), and it displayed uniformly nanocage structures approximately 12 nm in diameter through the TEM analysis (Figure 3c), akin to native ferritin [31].

It has been reported that FTH possesses ferroxidase activity, which can convert Fe^2+^ to Fe^3+^ and store it in the ferritin cage. When needed, Fe^3+^ can be converted back to Fe^2+^ and released [32]. The iron uptake kinetics of purified rhFTH were carried out in solutions with different concentrations of Fe^2+^, and the Fe^3+^ oxidized by ferritin was detected by UV absorbance at 310 nm. As shown in Figure 3d, the amounts of Fe^3+^ oxidized by the same concentration of rhFTH increased with the concentrations of Fe^2+^ in the solutions. An ICP-MS analysis also demonstrated that a single ferritin molecule could store 1159.3 ± 74.5 iron atoms when chelated with 5 mM Fe^2+^ solution (Figure 3e). These irons chelated by rhFTH could be released reversely into the solution in the presence of thioglycolic acid (Figure 3f). These in vitro characterizations of ferritin indicate that the purified rhFTH produced by *K. marxianus* indeed possesses ferroxidase activity, enabling it to oxidize Fe^2+^ to Fe^3+^, chelate, and store iron, which is consistent with native ferritin [2].

### 3.4. Analysis of Cytokine Secretion in RAW264.7 Mouse Macrophage Cell Line

**Figure 4:**
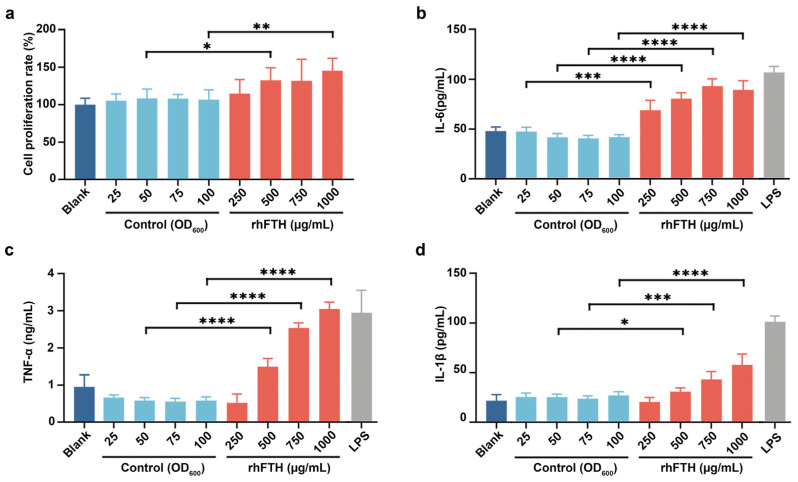
Effects of rhFTH-containing yeast cell lysates on the proliferation and cytokine secretion of RAW264.7 macrophage cell line after 24 h incubation with different treatments. Blank: culture medium; Control: host *K. marxianus* cell lysate without human ferritin; rhFTH: rhFTH contained in cell lysate; LPS: Lipopolysaccharide at 1 µg/L as the positive control. The biomass of the control host strain was adjusted to an OD_600_ equivalent to that of cell lysates containing 250, 500, 750, and 1000 µg/mL rhFTH. (**a**) Proliferation of the RAW264.7 cell line. (**b**) Secretion of IL-6 by the RAW264.7 cell line. (**c**) Secretion of TNF-α by the RAW264.7 cell line. (**d**) Secretion of IL-1β by the RAW264.7 cell line. Six replicates were set up for each group. Statistical analysis was performed using *t*-tests to determine significant differences, * *p* < 0.05, ** *p* < 0.01, *** *p* < 0.001, **** *p* < 0.0001.

As certain immunomodulatory foods can enhance the macrophage phagocytic activity, and promote the expression of cytokines, the biological function of rhFTH-containing yeast cell lysates was first evaluated by using RAW264.7 mouse macrophage cell line. The host *K. marxianus* cell lysates were used as a control. As shown in Figure 4, different concentrations of host *K. marxianus* cell lysates had no effect on the growth and cytokine secretion of the tested cells, consistent with the blank control (media only). However, when using the cell lysate containing 500 µg/mL ferritin nanoparticles, the proliferation of RAW264.7 mouse macrophage cells was significantly promoted (Figure 4a). Additionally, the cell lysate containing 500 µg/mL of ferritin nanoparticles significantly promoted the secretion of cytokines IL-6, TNF-α and IL-1β in RAW264.7 cells, showing a dose-dependent effect within the tested concentration range (Figure 4b–d). Compared to the positive control (LPS at 1 µg/L), the cell lysate containing 1000 µg/mL rhFTH promoted IL-6 secretion to 83%, TNF-α to 103%, and IL-1β to 57% of the positive control, respectively. This suggests that rhFTH nanoparticles contained in *K. marxianus* cell lysates have an immune-boosting effect by stimulating cytokine secretion.

### 3.5. Analysis of Biological Functions of rhFTH-Containing Cell Lysates in C. elegans Model

**Figure 5:**
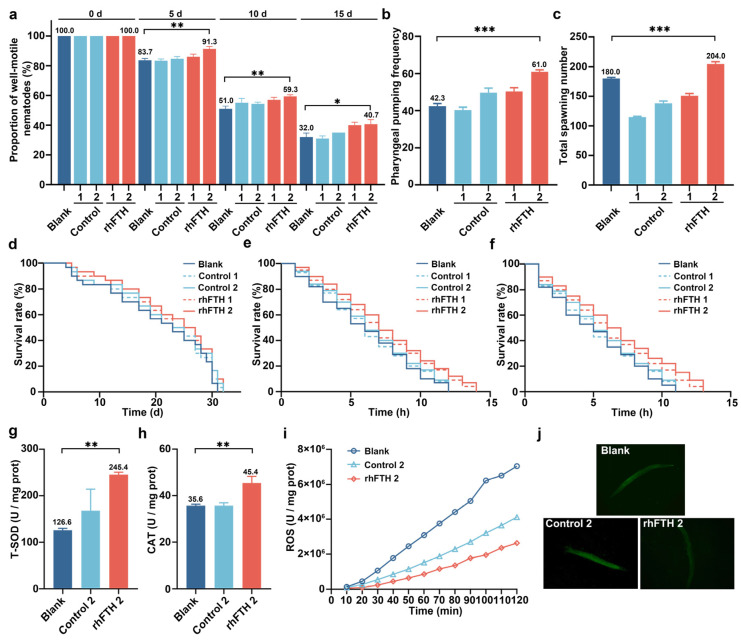
Analysis of biological function of rhFTH-containing yeast cell lysates on the *C. elegans* model. Blank: culture medium; Control 1: host *K. marxianus* cell lysate without human ferritin, and the biomass of the control host strain was adjusted to an OD_600_ equivalent to that of cell lysates containing 0.5 mg/mL rhFTH; Control 2: host *K. marxianus* cell lysate without human ferritin, and the biomass of the control host strain was adjusted to an OD_600_ equivalent to that of cell lysates containing 2.0 mg/mL rhFTH; rhFTH 1: 0.5 mg/mL rhFTH contained in cell lysate; rhFTH 2: 2 mg/mL rhFTH contained in cell lysate. (**a**) Proportion of well-motile nematode within 30 s at day 0, 5, 10, and 15. (**b**) Pharyngeal pumping frequency of nematodes within 1 min. (**c**) Total spawning number of nematodes. (**d**) Survival curves of nematodes under normal culture conditions (NGM, 20 °C). (**e**) Survival curves of nematodes under heat stress at 37 °C. (**f**) Survival curves of nematodes under H_2_O_2_-induced oxidative stress. (**g**) T-SOD activity. (**h**) CAT activity. (**i**) Quantization of ROS levels. (**j**) Analysis of the lipofuscin map. Statistical differences were analyzed using *t*-tests. * *p* < 0.05; ** *p* < 0.01; *** *p* < 0.001.

The biological function for lifespan extension of the rhFTH-containing yeast cell lysates was further tested in a *C. elegans* model. As shown in Figure 5a, the proportion of well-motile nematodes decreased as the culture time increased. On the 5th, 10th, and 15th day, the proportion of well-motile nematodes in the blank group was 83.7%, 51.0%, and 32.0%, respectively. In the group fed with 2 mg/mL rhFTH-containing cell lysate, the proportion of well-motile nematodes was 91.3%, 59.3%, and 40.7%, respectively, showing a 27.1% increase on the 15th day compared to the blank group. The pharyngeal pumping frequency of the nematode was also recorded (Figure 5b). The blank control group had 42.3 pumps per minute, while the group fed with 2 mg/mL rhFTH-containing cell lysate had 61.0 pumps per minute, a 44.1% increase. The total spawning number was also assessed, with the blank group producing 180.0 eggs and the group fed with 2 mg/mL rhFTH-containing cell lysate producing 204.0 eggs, representing a 13.5% increase (Figure 5c). In all test groups, the host yeast cell lysate showed no significant difference compared to the blank group.

A further analysis was conducted on the effects of rhFTH-containing yeast cell lysates on the lifespan of *C. elegans*. Under normal cultivation at 20 °C, the maximum lifespan of nematodes in all groups varied between 31 and 32 days with no significant difference (Figure 5d). However, under heat stress at 37 °C, the median survival time of the blank group was 6 h, with a maximum lifespan of 12 h. In the group fed with 2 mg/mL rhFTH-containing cell lysate, the median survival time was 7 h, with a maximum lifespan of 14 h, representing a 16.7% increase over the blank group (Figure 5e). Under H_2_O_2_ treatment, the median survival time was 5 h and the maximum lifespan was 11 h for the blank group, while the group fed with 2 mg/mL rhFTH-containing cell lysate had a median survival time of 6 h and a maximum lifespan of 13 h, representing increases of 20% and 18.2% compared to the blank, respectively (Figure 5f). Therefore, nematodes fed with rhFTH-containing cell lysate exhibited an approximately 20% increase in lifespan under both heat stress and H_2_O_2_ treatment.

Additionally, the T-SOD, CAT, and ROS levels in nematodes fed with 2 mg/mL rhFTH-containing cell lysate were analyzed. Compared to the blank group, T-SOD and CAT activities in the rhFTH group increased by 95.4% and 27.4%, respectively (Figure 5g,h), while ROS levels significantly decreased during the culture time (Figure 5i). An analysis of the lipofuscin map also showed clear antioxidant effects in the 2 mg/mL rhFTH group (Figure 5j). Thus, multiple indicators collectively demonstrate that feeding nematodes with 2 mg/mL rhFTH significantly enhances their antioxidant capacity, consistent with the extended lifespan results under heat and H_2_O_2_ stress conditions.

## 4. Discussion

In this study, we successfully expressed rhFTH in GRAS-grade *K. marxianus* [33], achieving a nanoparticle yield of 11 g/L in a 5 L fermenter, which is currently the highest yield reported for recombinant ferritin nanoparticles. The expressed rhFTH nanoparticles demonstrated both ferroxidase activity and the capability for iron storage. Previous studies using *K. marxianus* have also achieved high yields for other nanoparticle vaccines, such as PCV2 virus-like particles [23] and IBDV virus-like particles [25], surpassing yields using other expression systems including insect and yeast systems. These virus-like particles demonstrated immunogenicity. The successful expression of functional rhFTH in this study further suggests that *K. marxianus* may have inherent advantages for the expression of recombinant functional nanoparticle proteins. The rapid growth rate, broad growth temperature range, and high biomass of *K. marxianus* make it a favorable candidate for industrial production [34], likely due to its abundant energy supply and material metabolism [35]. This also indicates the potential of *K. marxianus* as an emerging food-safe industrial yeast, poised to play a significant role in future precision fermentation research and nutritional product manufacturing.

Encouragingly, the biological functionality of rhFTH-containing yeast cell lysate exhibited immune-boosting functions, while the yeast cell lysate alone did not show similar effects. Specifically, the rhFTH promoted cytokine secretion at the cellular level (Figure 4b–d). The appropriate activation of macrophages to increase cytokine secretion represents boosted immunity [36,37]. Several pathways, including NF-κB, AMPK, and PI3K-Akt, are known to mediate cytokine secretion [38]. Qu et al. reported that recombinant *Helicobacter pylori* ferritin, prepared through the baculovirus expression vector system, promoted the IL-6 and TNF-α secretion through initiation of the NF-κB pathway [39]. Additionally, Ruddell et al. found that ferritin activates an iron-independent signaling cascade involving Tim-2-independent PI3-kinase phosphorylation, PKCζ, and p44/p42-MAPK via the activation of the NF-κB pathway, resulting in p50/p65-NF-κB activation and the markedly enhanced expression of IL-1β [40]. Although the activation pathways of cytokines differ, the increased secretion of cytokines represents strengthened immunity [41,42]. RAW264.7 macrophage cell line is a recognized model for research of cytokine secretion and immunomodulation [43,44]. In our study, we found that rhFTH expressed in *K. marxianus* indeed promoted the secretion of IL-6, TNF-α, and IL-1β in the RAW264.7 macrophage cell line, indicating an immune-boosting effect. Furthermore, in the *C. elegans* model, rhFTH enhanced nematode vitality, including increased motility, pharyngeal pumping, and spawning (Figure 5a–c), as well as an extended lifespan under heat and H_2_O_2_ stress (Figure 5e,f), suggesting a potential link to improved immunity. In the rhFTH-treated group, increased SOD and CAT and decreased ROS levels were observed (Figure 5g–i), suggesting that rhFTH enhanced the antioxidant capacity of nematodes, supporting the extended lifespan under oxidative stress. Research by Valentini et al. showed that overexpression of the endogenous ferritin gene Ftn-1 in *C. elegans* extended their lifespan under tert-butyl hydroperoxide (t-BOOH) condition [45], supporting our findings. The biological functions exhibited by rhFTH may be attributed to different molecular mechanisms, and each specific mechanism requires further investigation. Regardless of the molecular mechanisms, the biological functions exhibited by rhFTH provide a scientific basis for the development of ferritin-based products.

The rhFTH nanoparticles expressed in *K. marxianus* not only demonstrate a similar size to native human ferritin nanoparticles but also exhibit ferroxidase activity, converting Fe^2+^ to Fe^3+^ and chelating the iron within the ferritin nanocage. Recombinant ferritin nanoparticles have been recognized as a versatile platform for medical applications [46], including encapsulating drugs for targeting tumor therapy [47], carrying imaging agents for tumor visualization [48], and displaying native-like antigens from human viral pathogens for vaccine immunization [49]. Despite the extensive exploration of recombinant ferritin-based technology, studies on the biological functions of recombinant ferritin itself are limited. The existing research, along with our findings, confirms that recombinant ferritin can promote cytokine secretion and boost immunity. This immune-boosting effect of recombinant ferritin further supports its potential use for adjuvant action in the development of ferritin-based nanomedicine and vaccine clinical applications. It also suggests that recombinant ferritin itself could be developed as a health product.

## 5. Conclusions

In conclusion, rhFTH expressed in *K. marxianus*, with a high yield of 11 g/L, the highest yield reported to date, is capable of efficient self-assembly into nanocages while demonstrating ferroxidase activity and iron-chelation properties. The expressed rhFTH demonstrated immune-boosting properties, enhanced vitality, and extended lifespan under stress conditions. These findings suggest that rhFTH from *K. marxianus* provides a valuable source of ferritin nanoparticles for developing ferritin-based products, such as tumor-targeted drugs, vaccines, and nutritional health foods. In product applications, the intrinsic biological functions of ferritin will augment the efficacy of ferritin-based products. Ferritin-based nutritional products will enrich the market for nutritional health products, providing significant benefits for human health. Additionally, GRAS-grade *K. marxianus* is reaffirmed as an ideal, food-safe industrial strain for precision fermentation.

## Data Availability

The data presented in this study are available upon request from the corresponding author.
